# Use of a Biologically Transparent Illumination Guide during Secondary Thoracoscopic Posterior Tracheopexy for Severe Tracheomalacia after Esophageal Atresia Repair: A Case Report

**DOI:** 10.70352/scrj.cr.26-0413

**Published:** 2026-08-27

**Authors:** Shoichi Tsuzaka, Kyoichi Deie, Motoki Ebihara, Rina Matsuda, Shotaro Taki, Shoko Ogawa, Itsuki Naya, Yoshihiro Hirata, Koichi Mizuta, Eito Imoto, Hiroshi Kawashima

**Affiliations:** 1 Department of Pediatric Surgery, Saitama Children’s Medical Center, Saitama, Saitama, Japan; 2 Department of Transplant Surgery, Saitama Children’s Medical Center, Saitama, Saitama, Japan; 3 Department of Neonatology, Saitama Children’s Medical Center, Saitama, Saitama, Japan

**Keywords:** tracheomalacia, esophageal atresia, posterior tracheopexy, biologically transparent illumination guide, redo surgery

## Abstract

**INTRODUCTION:**

Secondary thoracoscopic posterior tracheopexy (PT) for severe tracheomalacia (TM) following esophageal atresia (EA) repair is challenging because of postoperative adhesions and scarring. Meticulous dissection is required to separate the esophagus from the trachea, increasing the risk of iatrogenic injury. We report the first use of a biologically transparent (BT) illumination guide, Tumguide (Otsuka Pharmaceutical Factory, Tokushima, Japan), to assist in identifying the course of the esophagus during secondary thoracoscopic PT.

**CASE PRESENTATION:**

A 5-month-old infant with a history of thoracoscopic EA repair complicated by an anastomotic leak and stricture developed severe TM requiring surgical intervention. Because dense adhesions were anticipated, the Tumguide was activated in blinking mode and inserted after induction of general anesthesia to facilitate identification of the esophagus. Intraoperatively, the normal anatomical planes within the mediastinum were completely obliterated by severe fibrosis. However, the blinking BT red light was clearly visible through the thick scar tissue, helping clarify the overall orientation of the esophagus; a bronchoscope was also used as appropriate to transilluminate the tracheal wall from within, helping delineate the tracheal course. This combined visual guidance facilitated safe adhesiolysis and craniocaudal dissection, enabling successful fixation of the posterior membranous trachea to the anterior longitudinal ligament. The patient’s respiratory status improved markedly, and the patient remained stable in room air without symptoms 6 months postoperatively.

**CONCLUSIONS:**

The use of a BT illumination guide may serve as a useful and simple adjunct during complex redo surgeries such as secondary thoracoscopic PT. By helping delineate the course of the esophagus, it may improve spatial awareness and facilitate safe dissection even when normal anatomical planes are obliterated by dense adhesions.

## Abbreviations

BT
: biologically transparent
EA
: esophageal atresia
HFNC
: high-flow nasal cannula
LED
: light-emitting diode
PT
: posterior tracheopexy
TM
: tracheomalacia

## INTRODUCTION

TM, also known as tracheobronchomalacia, is a common complication in patients with EA. TM often causes severe respiratory symptoms such as cyanotic spells, recurrent pneumonias, and difficulty weaning from positive-pressure ventilation.^1–3)^ Thoracoscopic PT has emerged as an effective surgical approach for directly addressing the dynamic collapse of the posterior membranous trachea that characterizes TM in this population.^1,4,5)^ Specifically, PT involves suturing the flaccid posterior tracheal membrane to the anterior longitudinal ligament (or prevertebral fascia), thereby preventing dynamic airway collapse during expiration.^2,5)^

However, secondary PT performed after primary EA repair can be highly challenging. Postoperative adhesions and scarring make redo esophageal and airway surgery difficult and hazardous.^1,6)^ Meticulous sharp dissection is required to separate the esophagus from the trachea and access the anterior longitudinal ligament, increasing the risk of iatrogenic injury to the airway, esophagus, and recurrent laryngeal nerves.^1,6)^ Recently, the Tumguide (Otsuka Pharmaceutical Factory, Tokushima, Japan), a device that transmits BT red light, has been introduced into clinical practice.^7)^ Although originally designed to verify gastric tube positioning without radiation exposure, its surgical applications, including the present off-label use as an intraoperative navigational adjunct, are gradually expanding.^7,8)^ To our knowledge, this is the first reported use of a BT illumination guide during secondary thoracoscopic PT to help identify the course of the esophagus and facilitate adhesiolysis within a densely scarred mediastinum.

## CASE PRESENTATION

The patient was an infant born at 34 weeks and 0 days of gestation via an emergency cesarean section because of fetal bradycardia. The birth weight was 2140 g, with Apgar scores of 7 at 1 minute and 8 at 5 minutes. Immediately after birth, a nasogastric tube could not be advanced, and radiography revealed a coiled tube, leading to the diagnosis of EA. On day 3 of life, thoracoscopic repair of the EA was performed. The operative time was 3 hours and 8 minutes, with blood loss of 6 mL. Postoperatively, a minor anastomotic leak was detected on esophagography and was managed conservatively. However, the patient subsequently exhibited poor feeding, and esophagography at 1 month of age revealed an anastomotic stricture. Balloon dilation was performed twice, at 1 and 2 months of age, using a maximum balloon diameter of 10 mm and a pressure of 4 atm; this successfully improved the stricture.

**Fig. 1:**
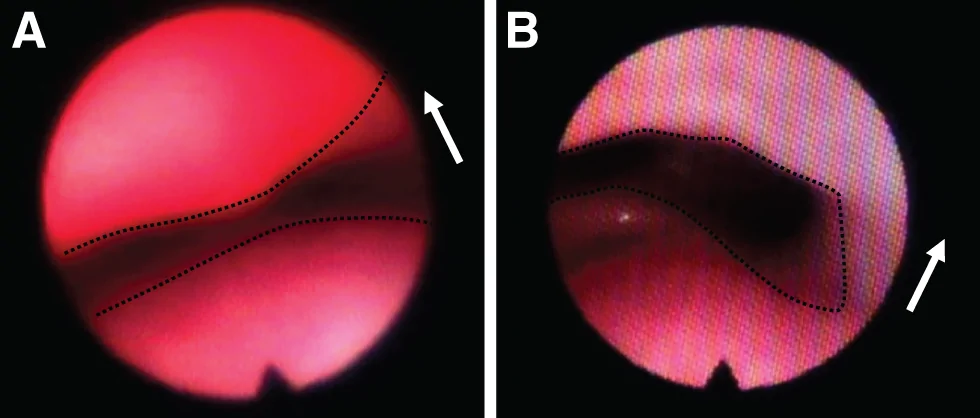
Bronchoscopic findings. (**A**) Preoperative bronchoscopic view showing severe TM, with an estimated expiratory airway collapse of approximately 77%. (**B**) Bronchoscopic view immediately after PT, showing marked expansion of the tracheal lumen, with the residual expiratory airway collapse reduced to approximately 27%. The dotted lines indicate the tracheal wall, and the tips of the white arrows indicate the anterior wall. PT, posterior tracheopexy; TM, tracheomalacia

**Fig. 2:**
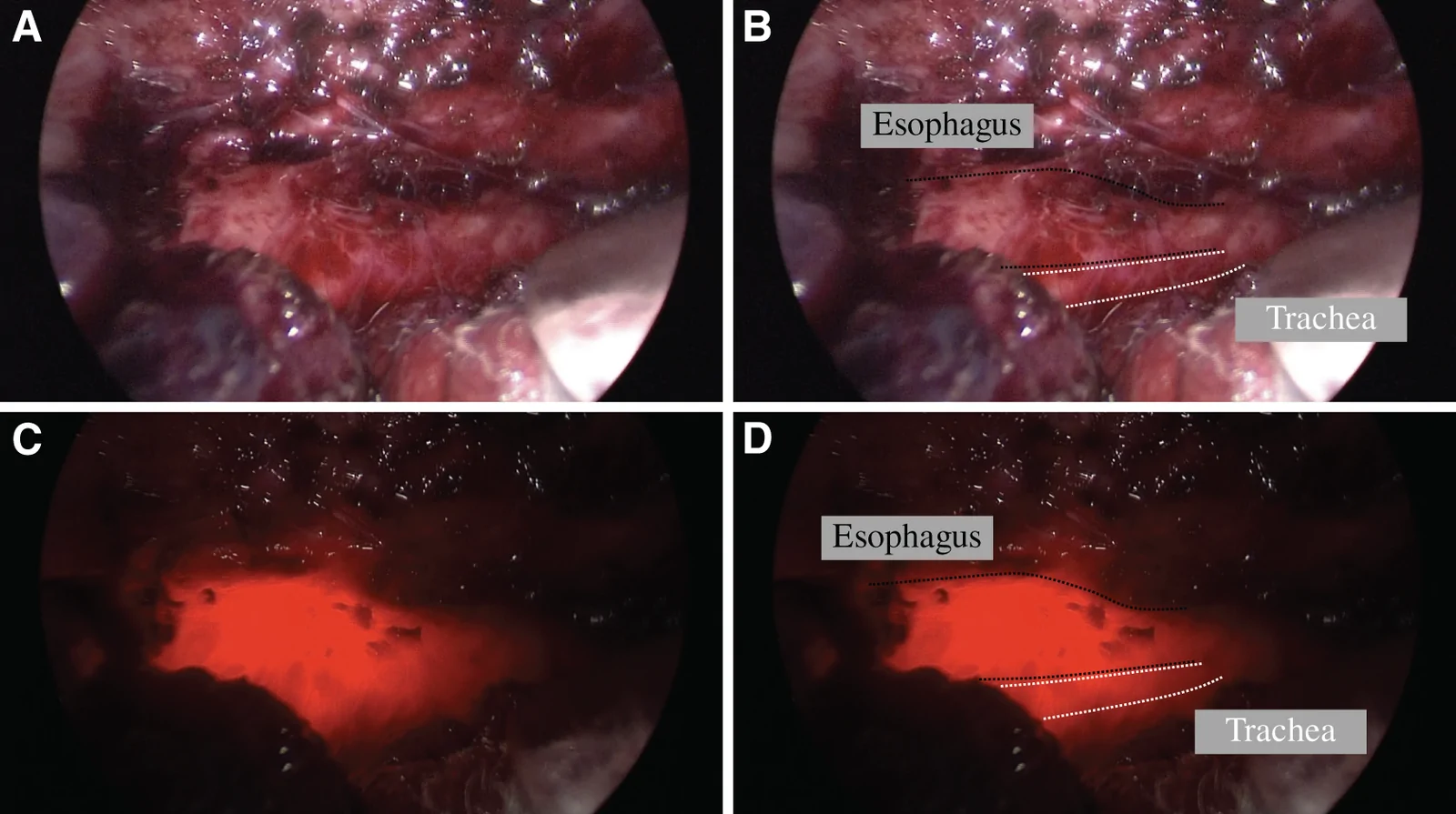
Intraoperative thoracoscopic views demonstrating the utility of the Tumguide. (**A**) Conventional view. The esophageal wall is difficult to identify because of severe adhesions. (**B**) The same view as in (**A**). The presumed course of the esophagus, estimated from Tumguide-guided transillumination, is outlined by black dotted lines, and the presumed course of the trachea, estimated from bronchoscopic transillumination, is outlined by white dotted lines. (**C**) View with Tumguide transillumination. Although the precise boundary remains indistinct, the overall orientation and course of the esophagus can be more readily appreciated through the scar tissue. (**D**) The same view as in (**C**). The esophageal and tracheal outlines, estimated from Tumguide and bronchoscopic transillumination findings, respectively, rather than from a clearly delineated anatomical boundary, are shown. Tumguide, Otsuka Pharmaceutical Factory, Tokushima, Japan

Regarding the respiratory status, the patient required continuous respiratory support with an HFNC following the initial EA repair. At 2 months of age, the patient experienced oxygen desaturation, and subsequent bronchoscopy confirmed severe TM, with an estimated expiratory airway collapse of approximately 77% (**Fig. 1A**). Respiratory management was transitioned to nasal continuous positive airway pressure with a positive end-expiratory pressure of 8–10 cmH_2_O. Although conservative management aimed at symptomatic improvement through weight gain was attempted, oral feeding exacerbated the TM symptoms, making it difficult to increase the feeding volume. Consequently, thoracoscopic PT was indicated at 5 months of age. Because of the patient’s history of an anastomotic leak and subsequent balloon dilations, severe dense adhesions surrounding the esophagus and trachea were anticipated. The Tumguide (Otsuka Pharmaceutical Factory) cannot be reactivated once turned off; therefore, it was activated in blinking mode and inserted into the stomach by the anesthesia team after induction of general anesthesia, in the same manner as a standard nasogastric tube. This was intended to facilitate identification of the esophagus and its relationship to the surrounding structures. The patient was placed in a near-prone position with a slight elevation of the right hemithorax, and a right thoracoscopic approach was performed. Ports were inserted through the same incisions used for the previous EA repair, and an artificial pneumothorax was established at 3 mmHg. Intraoperatively, after adhesiolysis between the lung and pleura, the mediastinal pleura was opened, revealing severe dense adhesions within the mediastinum with obliteration of the normal anatomical planes. Although the precise boundary between the esophagus and trachea remained indistinct because of severe fibrosis, the overall orientation and course of the esophagus could be more clearly appreciated by appropriately adjusting the position of the preoperatively placed Tumguide from the stomach into the esophagus. This intraoperative adjustment was performed by the anesthesia team at the surgeon’s request, without compromising the sterile surgical field. Notably, its blinking BT red light was clearly visible through the thick scar tissue without the need to dim the thoracoscopic illumination. To further delineate the tracheal course, a flexible bronchoscope was also inserted into the trachea, and its light source was used, as appropriate, to transilluminate the tracheal wall from within. By using the bronchoscope to delineate the tracheal outline and the Tumguide to delineate the esophageal outline, recognition of both structures was facilitated despite the densely fibrotic mediastinum, contributing to safer and smoother dissection (**Fig. 2**). Guided by this transillumination, the esophagus and trachea were dissected cranially and caudally to obtain adequate exposure of the posterior trachea and anterior longitudinal ligament. The membranous portion of the trachea (specifically, an approximately 2-cm segment extending cranially from immediately above the tracheal bifurcation) was then sutured and fixed to the anterior longitudinal ligament using 5 interrupted nonabsorbable sutures. Bronchoscopy, which had been used as needed during the dissection to help delineate the tracheal outline, subsequently confirmed expansion of the tracheal lumen, with the residual expiratory airway collapse reduced to approximately 27%, and the procedure was concluded (**Fig. 1B**). The total operative time was 4 hours and 39 minutes, with an estimated blood loss of 10 mL. The Tumguide remained active from insertion until the conclusion of the procedure, corresponding to a total device runtime of approximately 4 hours and 39 minutes, which was within the manufacturer-specified battery duration of approximately 5 hours.

The postoperative course was uneventful. The patient was extubated on POD 7, and enteral feeding via a nasogastric tube was initiated on POD 6. Although initiation of oral feeding was delayed until POD 33 because of decreased swallowing function, feeding no longer exacerbated the TM symptoms, allowing gradual advancement of nutritional intake. Although the patient initially required HFNC respiratory support after extubation, this was successfully weaned. At 6 months postoperatively, the patient remained stable on room air without respiratory symptoms.

## DISCUSSION

Secondary PT is an effective treatment option for severe TM associated with EA, but it requires extensive mobilization of the esophagus to access the anterior spinal ligament.^4)^ In patients with a history of primary EA repair, particularly those complicated by anastomotic leaks or recurrent strictures requiring dilation, the normal anatomical planes in the mediastinum are often obliterated by dense fibrotic scar tissues.^6)^ This scarring significantly increases the risk of iatrogenic injury to the esophagus, trachea, and recurrent laryngeal nerves during redo surgery.^6)^ Therefore, a technique that enables safe and continuous identification of the esophagus is highly desirable.

In this case, we used the Tumguide (Otsuka Pharmaceutical Factory), a BT red LED illumination system, together with a bronchoscope used as appropriate, enabling transillumination of the esophagus and trachea, respectively, during the dissection phase. The 660-nm red light has high tissue penetration capability, allowing transillumination through soft tissues over a short distance.^8,9)^ This is because wavelengths in the red-to-near-infrared spectrum (600–1300 nm) fall within the “optical window” of biological tissues, where absorption by water and hemoglobin is relatively low, thereby increasing tissue penetration depth.^10)^ Originally, this device was developed to verify the correct intragastric placement of nasogastric or orogastric tubes without radiation exposure, and its utility and safety have been well documented even in pediatric patients.^7,9)^ Recently, its applications have expanded into the surgical field. For example, it has been used to confirm the gastric tube positioning during laparoscopic gastrectomy to prevent accidental transection,^8)^ and Kanamori et al. reported the novel off-label use of this device to localize the lower esophageal blind end during delayed primary anastomosis for Gross type A long-gap EA.^7)^

However, to the best of our knowledge, this is the first report describing the use of a BT illumination guide during PT. Building on this concept, our case suggests the potential utility of this device in a much more hostile redo surgical environment. By inserting the optical fiber into the esophagus, the BT light transilluminated the esophageal wall from the inside outward. Interestingly, although the severity of the adhesions meant that the precise boundary between the esophagus and trachea remained somewhat unclear, the visual guidance provided by the red light allowed the surgical team to better appreciate the overall orientation of the esophagus. Awareness of the esophageal course within the dense scar tissue provided useful spatial guidance during dissection. Rather than relying solely on the tactile feedback from a standard gastric tube or on blind sharp dissection, continuous visualization of the esophageal orientation facilitated adhesiolysis and contributed to safer dissection, ultimately enabling safe mobilization of the esophagus and successful fixation of the posterior tracheal membrane. This combined use of transesophageal (Tumguide) and transtracheal (bronchoscope) transillumination, each applied as appropriate, allowed real-time recognition of both structures despite the severely fibrotic mediastinum, and we believe that it was a key element contributing to the safety and efficiency of the dissection in this case. Although PT is a highly beneficial procedure, it carries potential morbidities primarily related to extensive mediastinal dissection and repositioning of the esophagus.^1,11)^ Reported complications include recurrent laryngeal nerve injury, chylothorax, and esophageal complications such as dysmotility, stricture, and, rarely, perforation.^1,5)^ In this context, improved spatial awareness during dissection may help reduce the need for blind sharp dissection and potentially decrease the risk of iatrogenic injury. The real-time visual feedback provided by the Tumguide may be advantageous in this regard. Furthermore, this technique requires minimal setup and uses equipment already available for pediatric gastric tube verification.^9)^

However, this technique also has several limitations and technical considerations. First, the efficacy of transillumination and the clarity of organ boundaries may vary considerably depending on the severity of the adhesions, the thickness of the fibrotic tissue, and the patient’s individual anatomy. Second, although the Tumguide serves as a useful navigational adjunct by illuminating the esophageal lumen, it does not visualize other critical adjacent structures, such as the recurrent laryngeal nerve or thoracic duct. Therefore, it does not replace the fundamental requirement for meticulous surgical dissection based on a thorough understanding of the altered anatomy. Third, because the device cannot be reactivated once turned off, it must be activated preoperatively. Given that continuous operation is limited by a battery life of approximately 5 hours, careful time management is necessary, particularly during prolonged redo surgeries. In the present case, the total operative time was 4 hours and 39 minutes, and the device remained active throughout this period without depleting its battery, providing a real-world benchmark within this safety margin. Nonetheless, careful monitoring of the remaining runtime remains advisable during more prolonged redo surgeries. Finally, because this is a single case report, further experience and larger case series are needed to fully establish the clinical utility and safety of this approach in complex pediatric redo surgery.

## CONCLUSIONS

The use of a BT illumination guide (Tumguide; Otsuka Pharmaceutical Factory) may be a useful adjunct in complex redo surgery with severe adhesions, such as secondary thoracoscopic PT. By illuminating the orientation of the esophagus when normal anatomical planes are obliterated, it may facilitate adhesiolysis and improve spatial awareness during dissection. Although careful surgical dissection remains essential, this device may provide a simple adjunctive strategy for improving spatial awareness during complex redo surgery.
